# A Method of DTM Construction Based on Quadrangular Irregular Networks and Related Error Analysis

**DOI:** 10.1371/journal.pone.0127592

**Published:** 2015-05-21

**Authors:** Mengjun Kang, Mingjun Wang, Qingyun Du

**Affiliations:** 1 School of Resource and Environment Science, Wuhan University, Wuhan 430079, China; 2 Key laboratory of Geographic Information System, Ministry of Education, Wuhan University, Wuhan 430079, China

## Abstract

A new method of DTM construction based on quadrangular irregular networks (QINs) that considers all the original data points and has a topological matrix is presented. A numerical test and a real-world example are used to comparatively analyse the accuracy of QINs against classical interpolation methods and other DTM representation methods, including SPLINE, KRIGING and triangulated irregular networks (TINs). The numerical test finds that the QIN method is the second-most accurate of the four methods. In the real-world example, DTMs are constructed using QINs and the three classical interpolation methods. The results indicate that the QIN method is the most accurate method tested. The difference in accuracy rank seems to be caused by the locations of the data points sampled. Although the QIN method has drawbacks, it is an alternative method for DTM construction.

## Introduction

A digital terrain model (DTM) is an ordered set of sampled data points that represent the spatial distribution of various types of information on the terrain [1–3] and one of the most terrain information is elevation. When terrain information is one dimensional and only contains elevation, the model is equivalent to DEM (Digital Elevation Model). Obviously, DEM is a subset of DTM and the most fundamental component of DTM. In this paper, elevation is the only terrain information we interested in. DTMs can be generated from many sources of data: field surveys, existing topographic maps, stereo aerial photographs (at various scales) or satellite images [4]. The sampling pattern determines the method used for the construction of the DTM. There are two competing methods used to represent terrain: regular grids, or gridded DEMs, and triangulated irregular networks, or TINs. Scattered elevation points, which are one of the main data sources for DTMs, can be converted to TINs directly using linear interpolation [5] or converted into regularly gridded data points to construct gridded DEMs [6]. However, defects in the computational efficiency and accuracy of both methods have long been concerns.

The TIN method is considered optimal for constructing a DTM from irregularly located point elevations. TIN consists of a set of triangular facets defined by three edges, with each edge bounded by two vertices [7]. For most existing TIN-building methods, the task of the user is to select a set of points that best approximate the terrain surface according to a series of building criteria. For scattered point elevations, each point is used as a vertex for at least three triangles. The TIN method is known for its efficiency of data storage and the simplicity of its data structure for accommodating irregularly spaced elevation points [8]. However, the TIN method sometimes requires a large amount of data storage space to maintain the topology among each element, which lowers the speed of the algorithm. This is especially true for the wide range of landscapes [9] that are inevitable in some applications of DTM construction, such as hydrological analysis [10–12].

A gridded DEM represents the terrain surface by a regular square grid of sample elevation, gives a uniform intensity of sampling and is also known as the “altitude matrix” [13]. Gridded DEMs are used more frequently because of the wide availability and relatively low cost of data storage they provide. Prominent examples of gridded DEMs are the USGS 7.5-minute grid DEM and the SRTM data. Grid DEMs require much less computer storage allocation because only the elevation data at each point need to be stored; the x and y coordinates can be calculated from the starting point and grid spacing interval. In other DTM representation methods, these coordinates must be explicitly indicated [14]. Another advantage is that the topology is simple [15] because the neighbours of a selected point can be readily obtained from the “altitude matrix” to calculate the geomorphological parameters. Given this advantage, a wide variety of computer programs have been created based on gridded data. However, this method does have several disadvantages: (1) With a uniform grid spacing interval, grid DEMs tend to be redundant in smooth areas and are unable to portray small landscape features in areas where elevation varies rapidly over short distances; (2) With scattered elevation points (irregular spacing), the data must be converted into regularly spaced points by some method, such as TIN. According to error-propagation theory, DTM errors will propagate through this manipulation and manifest themselves in the final products [6, 16].

At present, neither method has been conclusively shown to be superior over a wide variety of terrain types [17]. Especially for scattered elevation point source data, the TIN method is not adopted for large areas of DTM construction, which increases the computation cost and lacks the efficiency necessary for some disciplines, such as hydrology [4]. Gridded DEMs are constructed from the spatial derivatives of point elevations without greatly considering the original point elevations, which makes the errors in the DTM larger. Even with recent improvements in computer hardware, there are still limitations in storage space and the computational cost of running the method. Considering these problems with DTM accuracy and computation efficiency, a new method that avoids the flaws of these two methods should be developed.

In this paper, a new method of DTM construction based on the quadrangular irregular network (QIN) is developed. The related research was first mentioned by Ferguson [18]. Hessing investigated automatic contouring with this method for small amounts of data [19]. Wu applied this method to relief generalization [20]. These studies attempted to establish a relationship between a QIN method and a DTM; however, the specific advantages of this method are not elaborated clearly, and the studies lack a comparative analysis of the accuracy of the DTM product.

A QIN method consists of a set of irregular quadrilateral meshes, where each mesh contains four vertices. All original scattered elevation points are regarded as quadrilateral vertices, and a series of new points is calculated by fitting a polynomial to the surrounding elevation points. All these vertices are organized in a topological matrix similar to the data structure of the gridded DEM. Therefore, the computer programs designed for gridded DEMs apply to QINs, ignoring the differences in the spatial interval.

To obtain the elevation (Z value) at a location on each mesh, two approaches are commonly employed [21]:

1. Each vertex is treated as the centre of a mesh. All locations on the mesh are assumed to have the same elevation as the central vertex.
2. A continuous surface is fitted to each mesh using adjacent vertices. The elevation of each point on the mesh can be calculated from the surface equation.

It is clear that the first method cannot approximate the real-world continuous surface well. To improve the accuracy of DTMs, a large number of studies have focused on DTM construction for interpolations, such as IDW, SPLINE [22], KRIGING [23], optimization methods based on theorems of surfaces [24, 25], fractal methods [26], and the Coons path method [16]. These interpolators can be constructed based on a quadrangular irregular network similar to the regular grid model because of their common topological structure. In this paper, we fit a polynomial surface to the irregular quadrilateral using bicubic interpolation, which preserves fine details better than common algorithms do. And an inter-comparison is drawn among this integral QIN method, other two interpolation methods (SPLINE and KRIGING) and TIN method, to analyse the simulation accuracy.

The rest of this paper is organized as follows. First, the construction rules of QIN is introduced, and then, the procedure of defining a smooth surface through a point matrix which is topologically equivalent to a planar rectangular grid is investigated. Second, a numerical test and a real-world example are employed to comparatively analyse the accuracy of QINs and that of the two classical interpolation methods SPLINE, KRIGING and TIN method. At last, discussion and conclusions are presented.

## Methods

### Principles of quadrangular irregular networks

The approximation of a surface by a set of points is important for DTMs. Several classical methods of solution have been proposed. A restriction of these methods is that the set of points must be rectangular sp aced (in the XY-plane). This method loosens the limitation of point distributions so that the point set is topologically equivalent to a planar rectangular grid [18].

Let *D* be the set of scattered elevation points *d*_*k*_ = *(x*_*k*_, *y*_*k*_, *z*_*k*_ *)*, *k* = 1,2,…,*K*. To obtain a point array *{P*_*n*,*m*_ *}*, *n* = 1,2, …, *N* and *m* = 1,2, …, *M*, which is topologically equivalent to an *N×M* planar rectangular grid, adjacent points must be connected by straight-line segments in the point set *D*. The resulting point array *{P*_*n*,*m*_ *}* divides the set *D* into “vertical subsets” *V*_*1*_, *V*_*2*_, *…*, *V*_*m*_, which satisfy the following principles [19]:

1. If *d*_*a*_ is a member of *V*_*m*_, and *d*_*b*_ is a member of *V*_*m+1*_, then *x*_*a*_ *<x*_*b*_.
2. If *d*_*a*_ and *d*_*b*_ are members of the same vertical subset, the line connecting (*x*_*a*_, *y*_*a*_) and (*x*_*b*_, *y*_*b*_) must form an angle with the y-axis that is less than *π/4*.
3. Each *V*_*m*_ must contain at least one member of *D*.
4. If *d*_*a*_ is a member of *V*_*1*_ or *V*_*M*_, it must also be on the boundary of the convex hull of *D*.

The set *D* is also divided into “horizontal” subsets *H*_*1*_, *H*_*2*_, *…*,*H*_*n*_, which follow the same principles:

1. If *d*_*a*_ is a member of *H*_*n*_ and *d*_*b*_ is a member of *H*_*n+1*_, then *y*_*a*_ *<y*_*b*_.
2. If *d*_*a*_ and *d*_*b*_ are members of the same horizontal subset *H*_*n*_, the line connecting (*x*_*a*_, *y*_*a*_) and (*x*_*b*_, *y*_*b*_ *)* must form an angle with the x-axis that is less than*π/4*.
3. Each *H*_*n*_ must contain at least one member of *D*.
4. If *d*_*n*_ is a member of *H*_*1*_ or *H*_*N*_, it must also be on the boundary of the convex hull of *D*.

For maximum computational efficiency, *π/4* is the best threshold: it keeps the vertical and horizontal point sets to a minimum and guarantees the characteristics of the topological matrix. If the angle is less than*π/4*, multiple new points will be produced. If the angle is more than*π/4*, the straight line segment connecting the known point to the points in the coincided area of the angle will be assigned to the horizontal vector and the vertical vector simultaneously.

**Fig 1:**
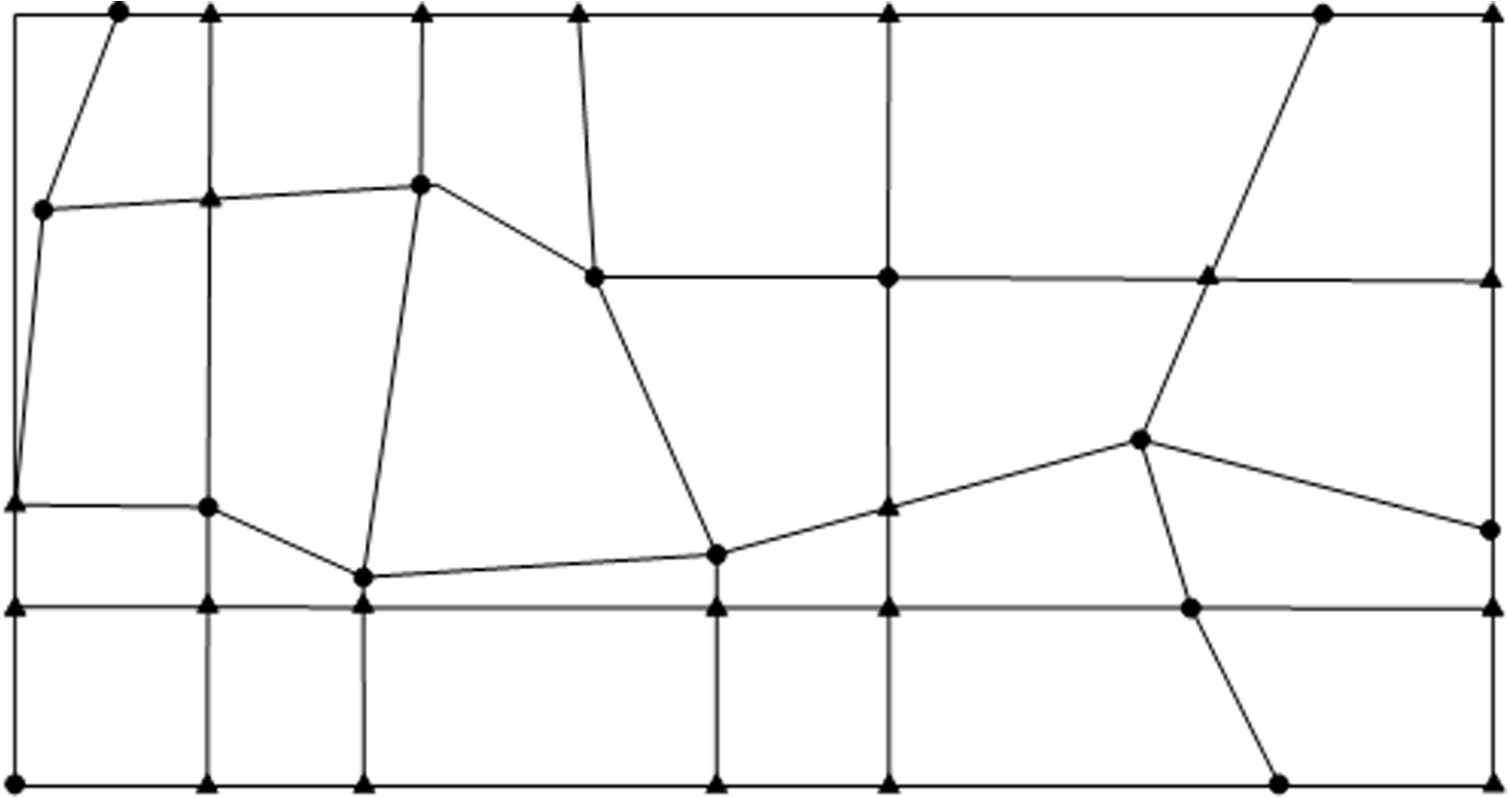
Quadrangular Irregular Network.

Let *I*_*n*,*m*_ = *H*_*n*_ *∩V*_*m*_. *I*_*n*,*m*_ indicates the intersection of one horizontal vector and one vertical vector; there is only, at most, one element belonging to two different vectors. There are two situations for *I*_*n*,*m*_: (1) If *I*_*n*,*m*_ is nonempty, call the element it contains *P*_*n*,*m*_; (2) If *I*_*n*,*m*_ is empty, a derivative point must be constructed that will satisfy conditions (1) and (2) for *H*_*n*_ and *V*_*m*_; call this point “new point”. Fig 1 show a quadrangular irregular network constructed from a sample data case. Black dots indicate original data and black triangular points indicate new points.

### Calculation of new points

After the above steps, the original data points are assigned to horizontal and vertical vectors and the values of *M* and *N*, which represent the number of the horizontal and vertical vector, respectively, are already clear. To determine the *x* and *y* coordinates of a new point *I*_*n*,*m*_, two linear equations involving line segments through the data points in *V*_*m*_ and *H*_*n*_ are used to calculate the intersection. For large numbers of scattered elevation points, the cost of computation is large and optimization should be considered; for example, an optimization that allows the calculation of a series of new points from two original data points to be performed only once.

To compute the entire quadrangular irregular network, the *z* values of new points must be obtained. A number of methods can be utilized, such as the inverse distance weighted (IDW) method, the rectangle-based blending method [27, 28], triangle-based blending methods [29], finite-element-based methods, Foley’s methods, and global-basis-function-type methods. For large numbers of elevation data points, to improve computational efficiency, storage requirements and ease of implementation, an inverse-distance-weighted method is employed in this paper. The basic function is given by [30, 31],

$$F\left(x,y\right)\;=\;\sum_{k=1}^{N}w_{k}\left(x,y\right)f_{k}/\sum_{k=1}^{N}w_{k}\left(x,y\right)$$ (1)

where *w*_*k*_(*x*, *y*) is the weight function, which has a variety of forms of expression, depending on data availability, and *f*_*k*_ is the function of known data.

To improve accuracy, two factors of the weighting function must be considered: distance and direction. As Tobler’s First Law [32] states, nearby points make greater contributions than distant points to any interpolated value; distant data points tend to influence the surface at higher order. For uneven data points, direction can be used to adjust the number of data points involved in the interpolation calculations. Considering these two factors, the interpolation method described below was proposed by Hessing [19]: Let (*x*_*n*,*m*_, *y*_*n*,*m*_) be a new point which must be interpolated, and let (*a*_*i*_,*b*_*i*_,*c*_*i*_), for *i* = 1, 2, …, *I*, be the data points. The circle centred at point (*x*_*n*,*m*_, *y*_*n*,*m*_) is subdivided into *4L* sections and the weights of the interpolation function are divided into *K* levels, for which *W*_*0*_ *> W*_*1*_ *>*, *…*, *> W*_*K*_ *> W*_*K+1*_ *= W*_*K+2*_ *=*, *…*, *= 0*. Let

$$d_{i}{}^{2}\;=\;{\left(a_{i}-x_{n,m}\right)}^{2}+{\left(b_{i}-y_{n,m}\right)}^{2}$$ (2)

and

$$\theta_{i}=tan^{-1}\left[\left(b_{i}-y_{n,m}\right)/\left(a_{i}-x_{n,m}\right)\right],$$ (3)

where *1⩽i⩽I*. For each *l*, *0⩽l⩽L-1*, four sectors are defined as follows:

$$\begin{array}{c} \pi l/2L\leq \theta <\pi /2+\pi l/2L,\\ \pi /2+\pi l/2L\leq \theta <\pi +\pi l/2L,\\ \pi +\pi l/2L\leq \theta <3\pi /2+\pi l/2L,\\ 3\pi /2+\pi l/2L\leq \theta <0\;\text{or}\;0\leq \theta <\pi l/2L. \end{array}$$

Let *J*_*i*,*l*_ be the number of data points (*a*_*j*_, *b*_*j*_, *c*_*j*_) such that *θ*_*i*_ and *θ*_*j*_ are in the same sector and d_j_^2^ < *d*_*i*_^2^. The weight function of point (*a*_*i*_, *b*_*i*_, *c*_*i*_) is described as.

$$W_{i}=\left(1/d_{i}{}^{2}\right)\sum_{l=0}^{L-1}\;W_{J_{i,l}}$$ (4)

For accuracy and efficiency of the interpolation algorithm, *W*_*0*_ = *1*, *W*_*1*_ = *1/2*, …, *W*_*K*_ = *W*_*K-1*_ */2* and *L* = *10*, *K* = *4* are the parameter values suggested by Hessing after a series of experiments. This method follows Tobler’s First Law and other derivative rules, such as giving relatively isolated data points more weight and giving clustered data points less weight to an even greater degree than isolated points. One important factor for interpolation is the sample strategy. In this method, *K = 4* determines the number of data points, which means that up to 5 data points can be involved in a computation. However, if the value of *d*_*i*_ is large, the contribution of each point is small, and errors may be introduced. Therefore, a threshold *R* for *d*_*i*_ should be used. Suppose *N* is the total number of data points and *A* is the area of the convex hull of all data points; then

$$R=\sqrt{10\left(\frac{A}{\pi N}\right)},$$ (5)

where *R* is defined as 10 times the average spacing of the data points. After these configurations, the interpolation can be easily implemented. New points interpolated from data points affect the accuracy of the entire QIN. A detailed error analysis will be presented in “Analysis” section.

### Construction of a bicubic surface

The bicubic surface method was originally developed by Ferguson in the 1960s for use in the car industry [18]. The basic idea of a Ferguson surface is to define a smooth surface through a point array whose arrangement is topologically equivalent to a planar rectangular grid, to extend the permission of point array distributions. The resulting solution is a smooth composite of parametric surface segments which is second order continuous. A Ferguson surface can be expressed by the following function:

$$S_{n,m}\left(u,v\right)=\left[X_{n,m}\left(u,v\right),Y_{n,m}\left(u,v\right),Z_{n,m}\left(u,v\right)\right]$$ (6)

where 0 ≤ *u* ≤ 1, 0 ≤ *v* ≤ 1

$$X_{n,m}\left(u,v\right)=\sum_{p=0}^{3}\sum_{q=0}^{3}\;a_{p,q}^{n,m}u^{p}v^{q},$$ (7)

$$Y_{n,m}\left(u,v\right)=\sum_{p=0}^{3}\sum_{q=0}^{3}\;b_{p,q}^{n,m}u^{p}v^{q},$$ (8)

$$Z_{n,m}\left(u,v\right)=\sum_{p=0}^{3}\sum_{q=0}^{3}\;c_{p,q}^{n,m}u^{p}v^{q}.$$ (9)

**Fig 2:**
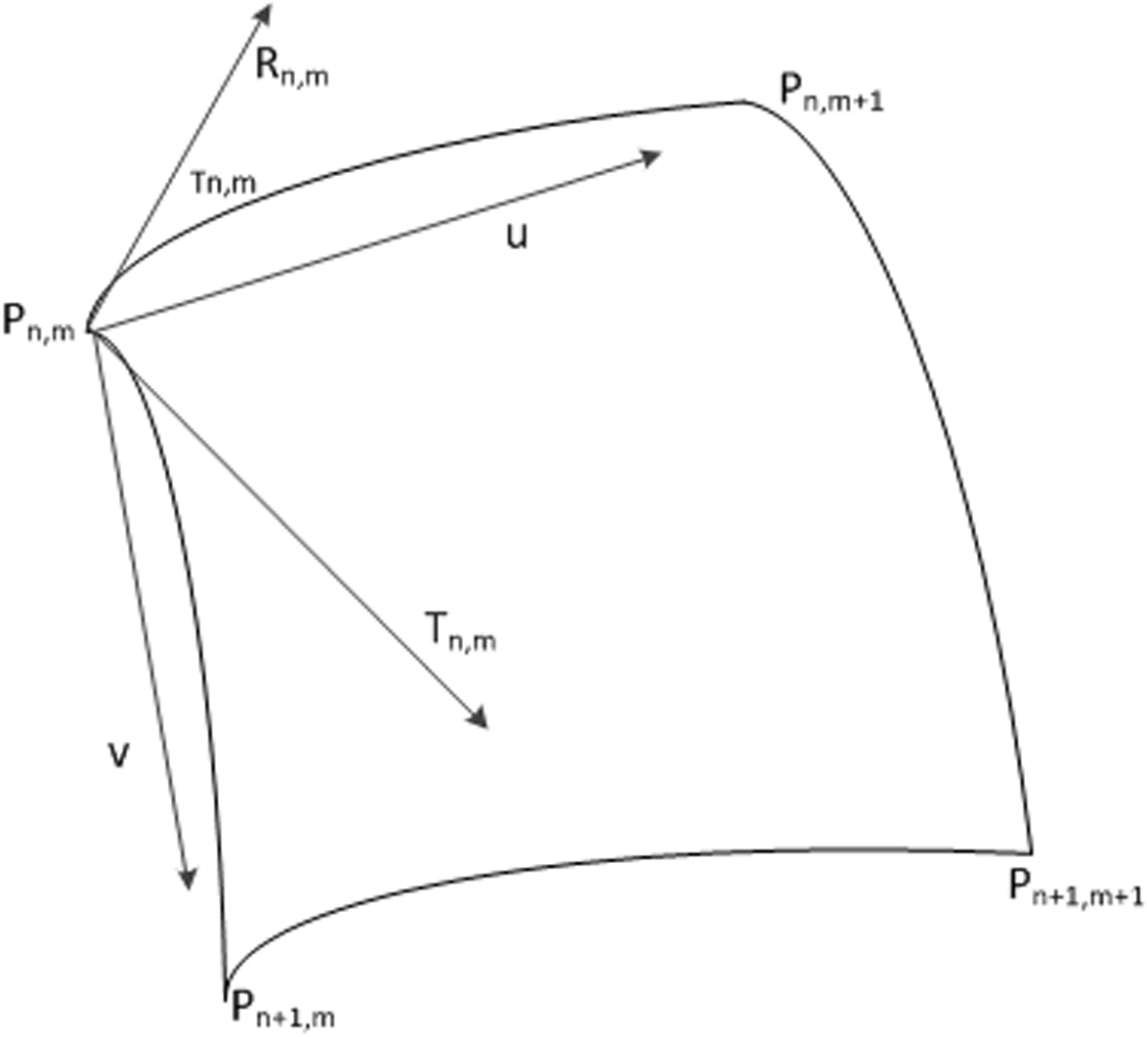
The quadrilateral with four corner points P_n,m_, P_n,m+1_, P_n+1,m_, P_n+1,m+1_ and the partial derivatives R_n,m_, T_n,m_ on data point P_n,m_.

There are thus 16 coefficients to determine. The Ferguson surface contains the four corner points of each quadrilateral bounded by the curves [*X*_*n*,*m*_(0, *v*), *Y*_*n*,*m*_(0, *v*)], [*X*_*n*,*m*_(*u*, 0), *Y*_*n*,*m*_(*u*, 0)], [*X*_*n*,*m*_(1, *v*), *Y*_*n*,*m*_(1, *v*)], and [*X*_*n*,*m*_(*u*, 1), *Y*_*n*,*m*_(*u*, 1)]. Therefore, the conditions can be obtained as follows (Fig 2):

$$S_{n,m}\left(u,v\right)=\{\begin{array}{r} P_{n,m}\;if\;u=v=0\\ P_{n,m+1}\;if\;u=1,v=0\\ P_{n+1,m}\;if\;u=0,v=1\\ P_{n+1,m+1}\;if\;u=v=1 \end{array}$$ (10)

Let *R*_*n*,*m*_ be the partial derivatives of *u* and *T*_*n*_,_*m*_ be the partial derivatives of *v*. To meet the second order continuous condition, additional conditions are also needed:

$$\frac{\partial S_{n,m}}{\partial u}\left(u,v\right)=\{\begin{array}{r} R_{n,m}\;if\;u=v=0\\ R_{n,m+1}\;if\;u=1,v=0\\ R_{n+1,m}\;if\;u=0,v=1\\ R_{n+1,m+1}\;if\;u=v=1 \end{array}$$ (11)

and

$$\frac{\partial S_{n,m}}{\partial v}=\{\begin{array}{r} T_{n,m}\;ifu=v=0\\ T_{n,m+1}\;if\;u=1,v=0\\ T_{n+1,m}\;if\;u=0,v=1\\ T_{n+1,m+1}\;if\;u=v=1 \end{array}$$ (12)

Therefore, the bicubic surface can be described as,

$$S_{n,m}\left(u,v\right)=UAW^{T}=UMBM^{T}W^{T}$$ (13)

where *A = MBM* ^*T*^, $B=\left[\begin{array}{ccc} P_{n,m} & P_{n,m+1} & \begin{array}{cc} R_{n,m} & R_{n,m+1} \end{array}\\ P_{n+1,m} & P_{n+1,m+1} & \begin{array}{cc} R_{n+1,m} & R_{n+1,m+1} \end{array}\\ \begin{array}{c} T_{n,m}\\ T_{n+1,m} \end{array} & \begin{array}{c} T_{n,m+1}\\ T_{n+1,m+1} \end{array} & \begin{array}{cc} \begin{array}{c} P_{n,m}^{u,v}\\ P_{n+1,m}^{u,v} \end{array} & \begin{array}{c} P_{n,m+1}^{u,v}\\ P_{n+1,m+1}^{u,v} \end{array} \end{array} \end{array}\right],$ $M=\left[\begin{array}{ccc} 2 & -2 & \begin{array}{cc} 1 & 1 \end{array}\\ -3 & 3 & \begin{array}{cc} -2 & -1 \end{array}\\ \begin{array}{c} 0\\ 1 \end{array} & \begin{array}{c} 0\\ 0 \end{array} & \begin{array}{cc} \begin{array}{c} 1\\ 0 \end{array} & \begin{array}{c} 0\\ 0 \end{array} \end{array} \end{array}\right],\;U=\left[u^{3}u^{2}u\;1\right],\;V=\left[v^{3}v^{2}v\;1\right]$ and ${\text{P}}_{\text{n},\text{m}}^{\text{u},\text{v}}={\frac{\partial^{2}\text{S}}{\partial \text{u}\partial \text{v}}|}_{\text{u}=\text{n},\text{v}=\text{m}}$. Eq 13 is called a Coons bicubic surface, which is difficult to compute because the mixed partial derivatives of the four corner points are difficult to determine. Therefore, a Ferguson surface is utilized, which sets the four mixed partial derivatives to 0; that is,

$$B=\left[\begin{array}{ccc} P_{n,m} & P_{n,m+1} & \begin{array}{cc} R_{n,m} & R_{n,m+1} \end{array}\\ P_{n+1,m} & P_{n+1,m+1} & \begin{array}{cc} R_{n+1,m} & R_{n+1,m+1} \end{array}\\ \begin{array}{c} T_{n,m}\\ T_{n+1,m} \end{array} & \begin{array}{c} T_{n,m+1}\\ T_{n+1,m+1} \end{array} & \begin{array}{cc} \begin{array}{c} 0\\ 0 \end{array} & \begin{array}{c} 0\\ 0 \end{array} \end{array} \end{array}\right]\cdot \text{s}$$ (14)

Now, to obtain all the coefficients, we focus on the calculation of *R*_*n*,*m*_ and *T*_*n*,*m*_ near the data point *P*_*n*,*m*_. A method that considers the distance-based weight was proposed by Hessing. Let

$$\begin{array}{c} E=\left(\frac{1}{D_{1}{}^{2}}\right)\left(P_{n,m}-P_{n,m-1}\right)+\left(\frac{1}{D_{2}{}^{2}}\right)\left(P_{n,m+1}-P_{n,m}\right)\;,\\ D_{1}{}^{2}={\left(x_{n,m}-x_{n,m-1}\right)}^{2}+{\left(y_{n,m}-y_{n,m-1}\right)}^{2} \end{array}$$

and

$$D_{2}{}^{2}={\left(x_{n,m+1}-x_{n,m}\right)}^{2}+{\left(y_{n,m+1}-y_{n,m}\right)}^{2}.$$

Thus *E = (E*_*x*_, *E*_*y*_, *E*_*z*_ *)* is the weighed sum of *P*_*n*,*m—*_ *P*_*n*,*m-1*_ and *P*_*n*,*m+1—*_ *P*_*n*,*m*_. Finally, the equations are complete: $R_{n,m}=\frac{\left(Min\left(x_{n,m}-x_{n,m-1},x_{n,m+1}-x_{n,m}\right)\right)}{E_{x}}$ and $T_{n,m}=\frac{\left(Min\left(y_{n,m}-y_{n,m-1},y_{n,m+1}-y_{n,m}\right)\right)}{E_{y}}$. After the above configurations, a second order continuous surface has been constructed that covers all the points, and each mesh of the surface is constructed by one quadrilateral. The surface represents the true terrain elevations and can be treated as data based on digital terrain analysis.

## Analysis

### Numerical test

#### The Peaks surface

**Fig 3:**
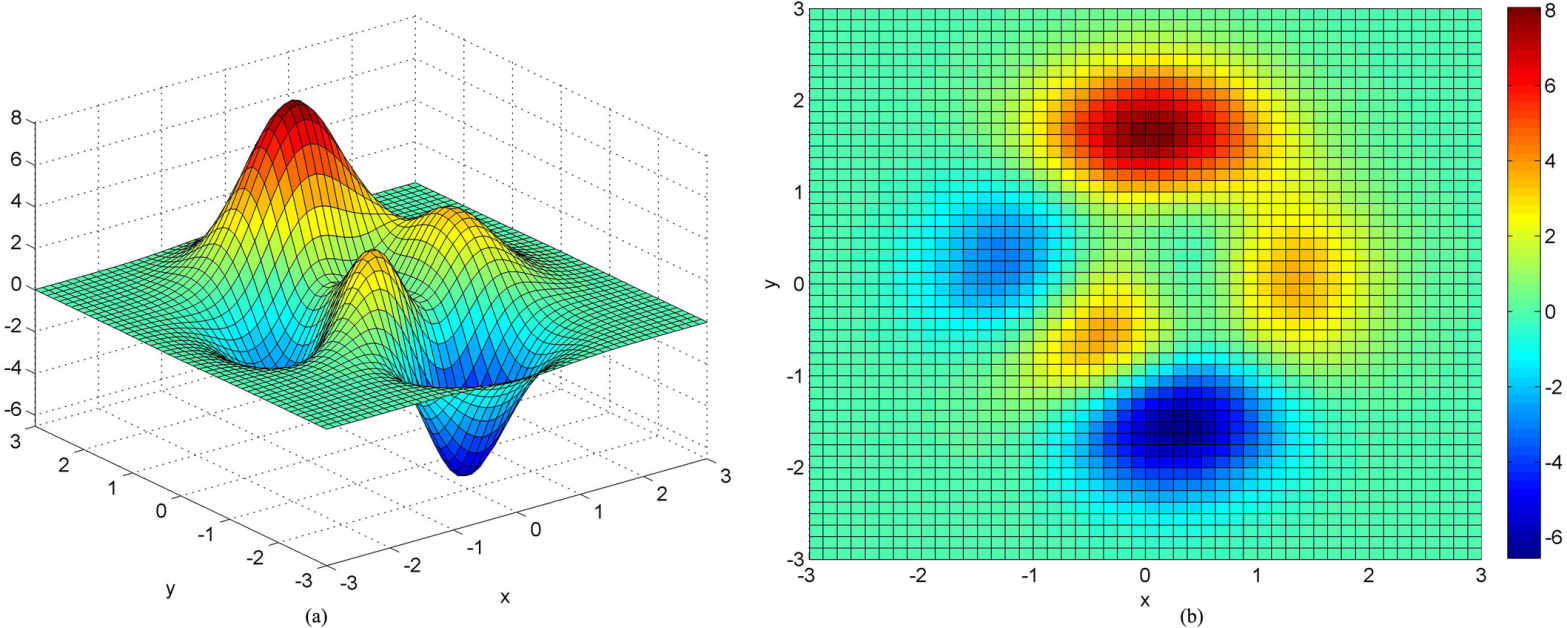
Peaks surface is function of three variables, obtained by translating and scaling Gaussian distributions, which is useful for DEM test. (a) 3D display (b) 2D display.

**Fig 4:**
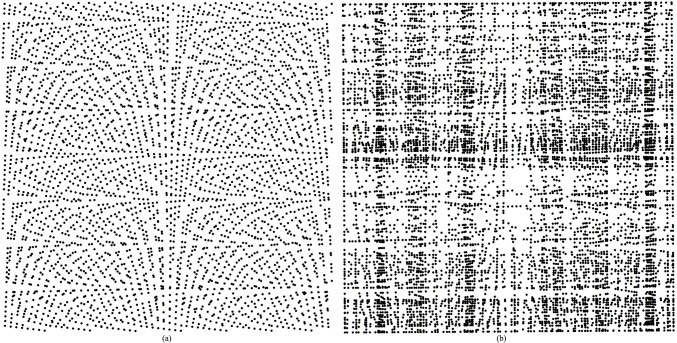
Distribution of both (a) the original data points and (b) the new ones.

**Fig 5:**
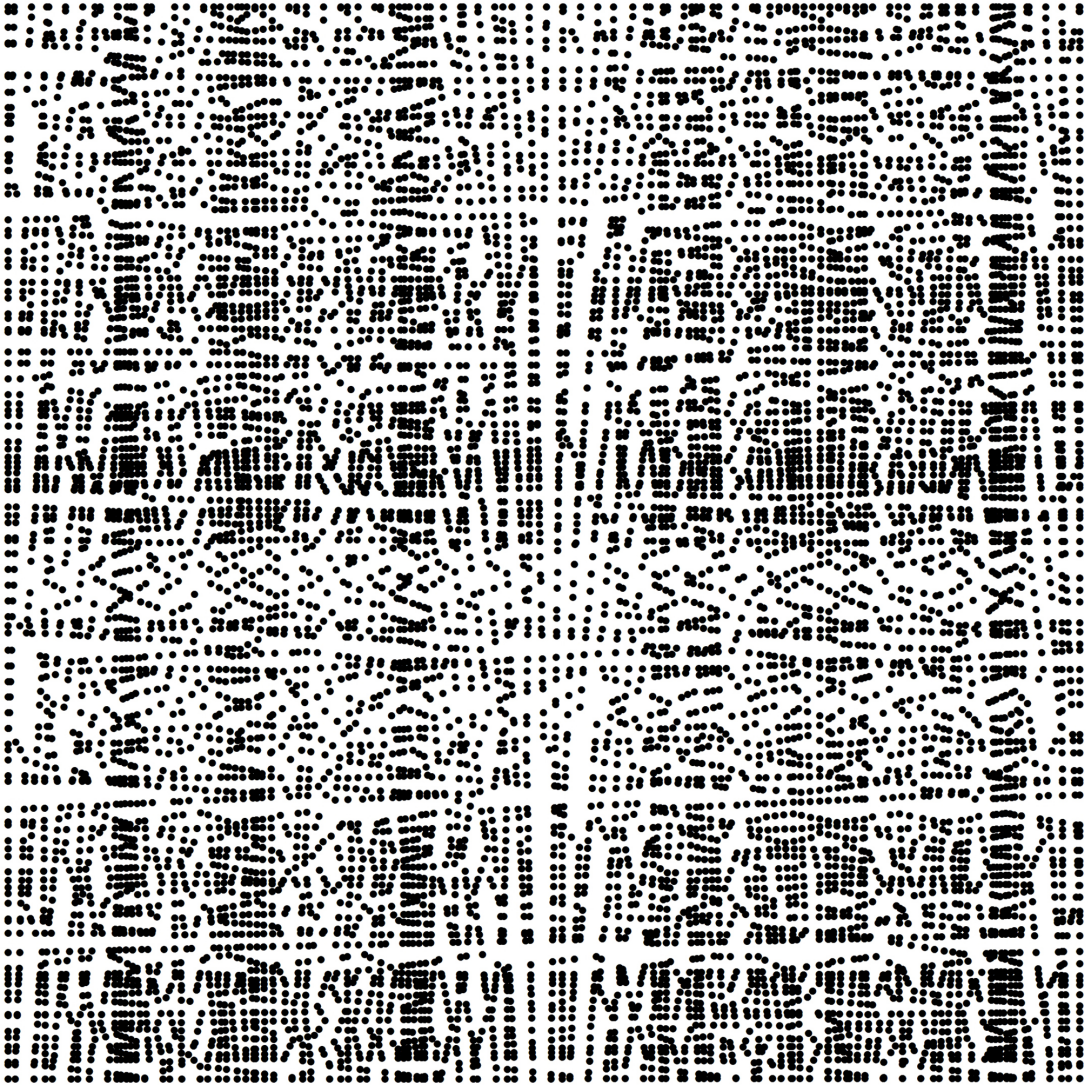
Distribution of all points.

**Fig 6:**
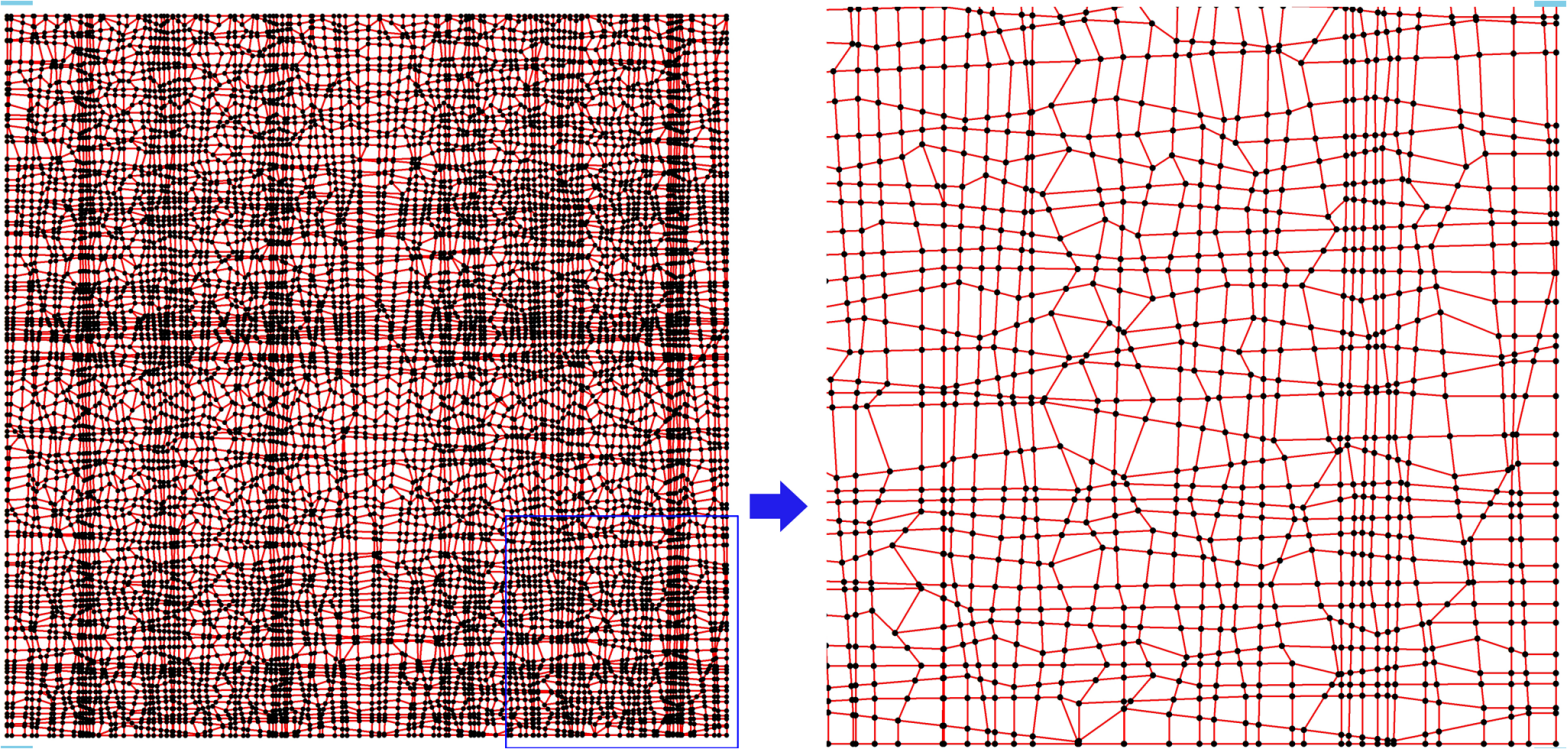
QIN constructed network and its magnified information.

**Fig 7:**
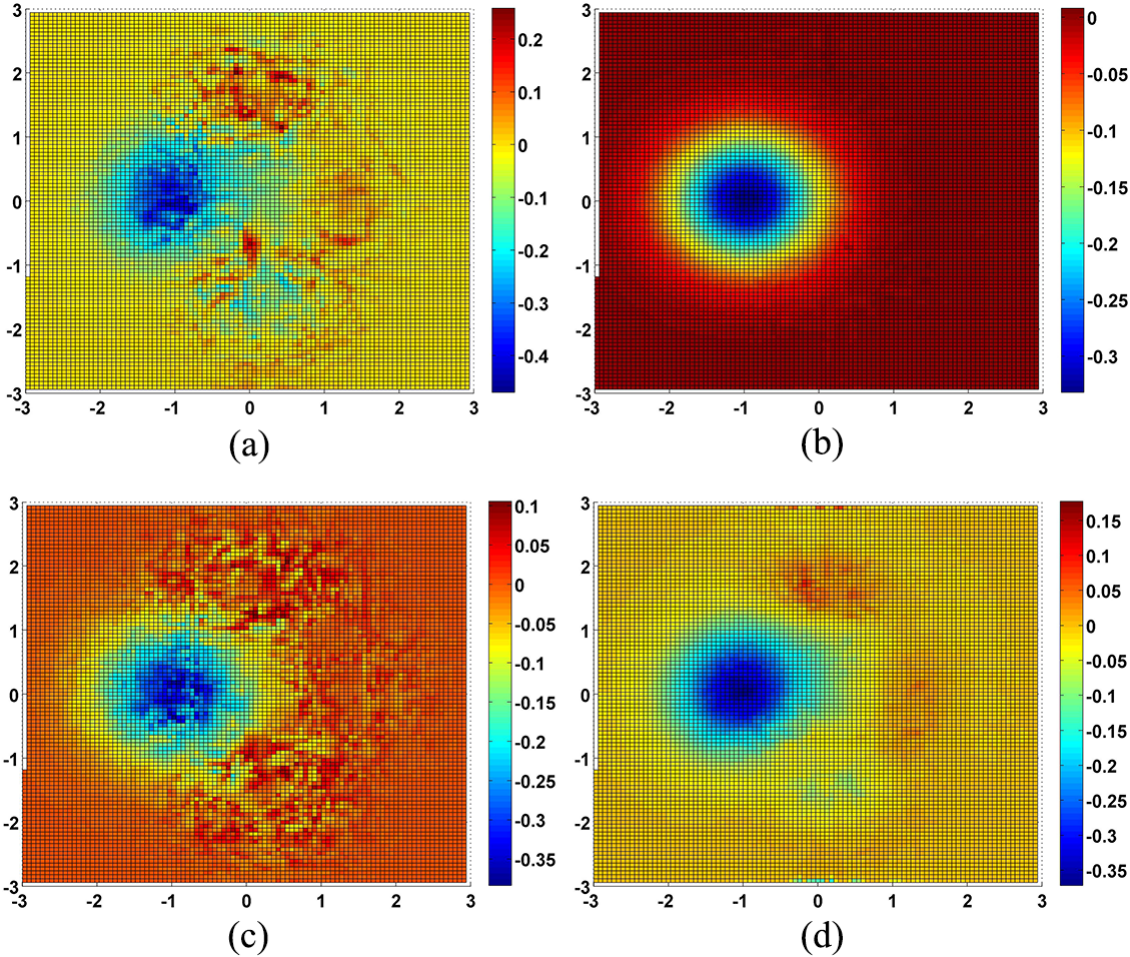
New points error distribution of simulations. (a) IDW, (b) KRIGING, (c) SPLINE, (d) TIN.

$$z=3\times {\left(1-x\right)}^{2}\times exp\left(\left[-x^{2}-{\left(y+1\right)}^{2}\right]\right)-10\times \left(\frac{x}{5}-x^{3}-y^{5}\right)\times exp\left(-x^{2}-y^{2}\right)-\frac{1}{3}\times exp\left(-{\left(x+1\right)}^{2}-y^{2}\right)$$ (15)

was used as a numerical test surface. The true value at each point can be determined by the equation to avoid uncertainty introduced by uncontrollable data errors present in real world data (Fig 3). The computational domains of *x*, *y* and *z* are [-3,3] by [-3,3] by [-6,6], respectively. 3600 points were randomly chosen as the original data points. The sample strategy is that the XY-plane was divided into 60×60 grids. In each grid, a point was sampled by calculating its *x* and *y* coordinates randomly; its *z* value was then calculated from the Peaks equation (Fig 4). The *x* and *y* values of 7308 new points were calculated from the intersection of two line segments, and the *z* values of these points were calculated by the Hessing IDW algorithm (Fig 5). After these processing steps, the 101×108 quadrangular irregular networks were constructed successfully. Fig 6 indicates the distribution of all data points, and Fig 7 indicates the quadrangular networks constructed by the Hessing rules.

In addition to the QIN method, there are some classical interpolation methods including KRIGING, SPLINE which are widely used in GIS applications. These two interpolation methods and TIN method were employed to comparatively analyse the errors of the QIN method. The spatial analyst and 3D analyst toolboxes in ArcGIS 10.0 were used to perform these three classical methods; all default parameters of the software were kept. For KRIGING interpolation, the ordinary option was selected, the semivariogram model was spherical, the search radius was variable and the maximum number of search points was 12. For SPLINE interpolation, the REGULARIZED option was used, the weight was 0.1 and the number of searched points was 12. For TIN method, the original data points were employed as the input feature class.

The domain of the Peaks surface was orthogonally divided into 10,000 (100×100) lattices, so there were 10,000 check points that were interpolated by the three classical methods to comparatively analyse the accuracy of the QIN method. The root mean-square error (RMSE) is expressed as [25]

$$RMSE=\sqrt{\frac{1}{100\times 100}\sum_{i=1}^{100}\sum_{j=1}^{100}\left(f_{i,j}-sf_{i,j}\right)},$$ (16)

where *sf*_*i*,*j*_ is the numerical result at the point (*x*_*i*_,*y*_*i*_) calculated by the Peaks equation and *f*_*i*,*j*_ is the interpolated value of the simulated surface at point (*x*_*i*_,*y*_*i*_).

The QIN construction process consists of two steps. First, the 7308 new data points were calculated by Hessing IDW methods. Second, the 10,000 check points were calculated by the bicubic method. Accuracy of all the new data points was calculated as Table 1 shows, KRIGING has the lowest RMSE value, and Hessing IDW has the highest RMSE value. The absolute maximum error and the standard deviation of Hessing IDW are also the highest of any method. In this step, KRIGING has the best accuracy, followed by SPLINE and TIN, and Hessing IDW performs worst. An error matrix [*E*_*i*,*j*_] for each method is constructed to express the spatial distribution of errors; the *E*_*i*,*j*_ are formulated as

$$E_{i,j}=Sf_{i,j}-f_{i,j}$$ (17)

where *f*_*i*,*j*_ is the true value of the surface and *Sf*_*i*,*j*_ is the simulated value of the surface at the point (*i*,*j*) [25]. Fig 7 indicates that these four methods have a relatively concentrated and negative error region, which is coloured blue in the figures; this area is concave in the Peaks surface. The error distribution of Hessing IDW and SPLINE is more complex, while that of the KRIGING is more uniform.

**Table 1:** RMSE and other information of new points employed simulation methods.

	Hessing IDW	SPLINE	KRIGING	TIN
**RMSE**	0.081907	0.072383	0.068443	0.073316
**ABS Max Error**	0.487149	0.414068	0.333527	0.376977
**ABS Min Error**	4.0724E-8	2.2394E-8	4.5896E-9	3.0097E-8
**Standard Deviation**	0.070117	0.063220	0.062895	0.065836

**Fig 8:**
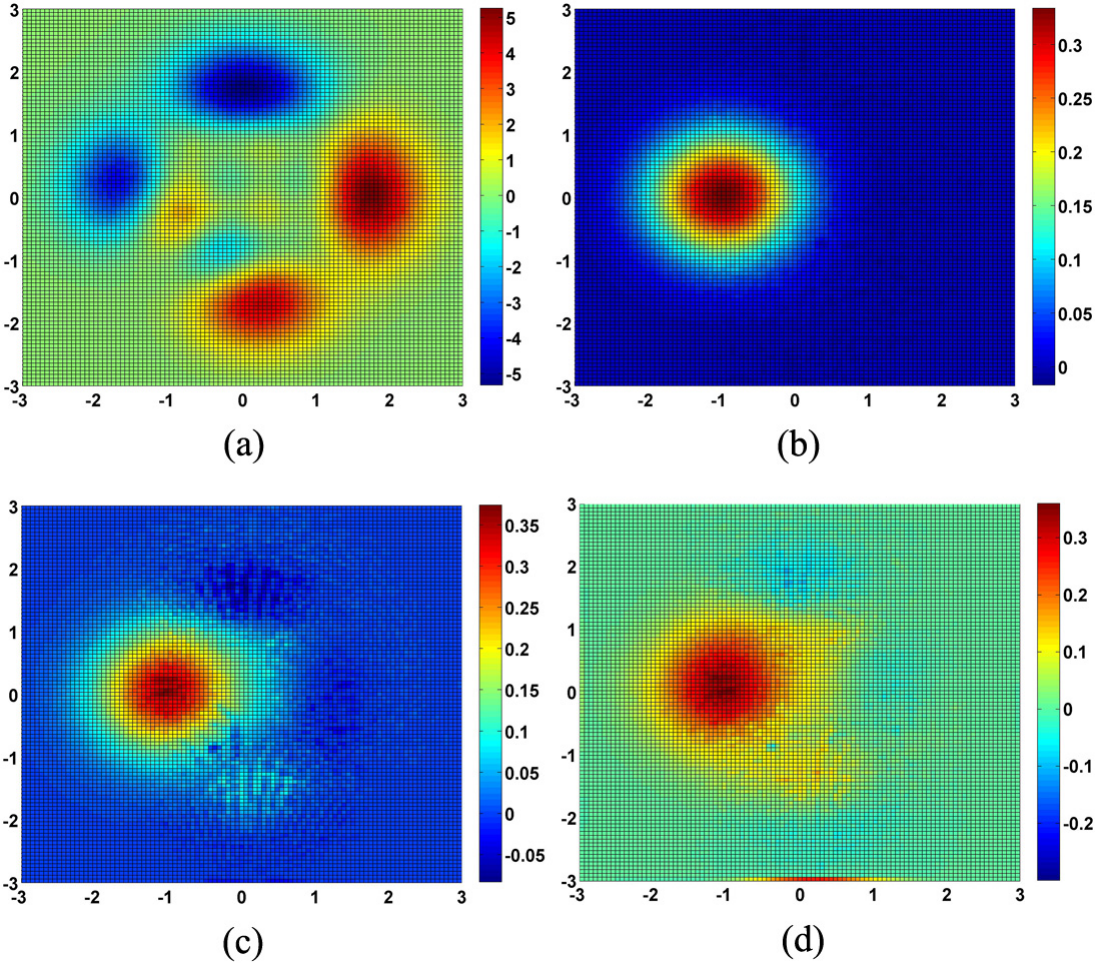
Check points error distribution of simulations. (a) QIN, (b) SPLINE, (c) KRIGING, (d) TIN.

Hessing IDW performs the worst in the first step, but this does not mean that the QIN method has a higher error, because this accuracy analysis is just about the new data points in the first step. With these new data points, the QIN meshes were constructed completely and the bicubic surface can be constructed based on the meshes. As Table 2 shows, the QIN method actually delivers the second best accuracy. SPLINE has the highest accuracy, and TIN performs the worst. Therefore, the overall accuracy ranking is different from that of the first step. With the increased number of points involved in the interpolation calculation, the accuracy of both the SPLINE and QIN methods improve. Fig 8 shows that the accuracy of QINs is greatly affected in areas with steep slopes; the Peaks surface has four such areas of high error. SPLINE and KRIGING show a concentrated area of high error, and overall, the simulated *z* value is lower than the true value. TIN also has a region of high error, but in other areas the result is closer to the true value.

**Table 2:** RMSE and other information of check points employed simulation methods.

	QIN	SPLINE	KRIGING	TIN
**RMSE**	0.06950	0.06897	0.07031	0.07381
**ABS Max Error**	0.07599	0.33389	0.37400	0.36035
**ABS Min Error**	3.6432E-8	3.8997E-9	4.4226E-10	3.8104E-8
**Standard Deviation**	0.01723	0.06272	0.06300	0.06401

**Fig 9:**
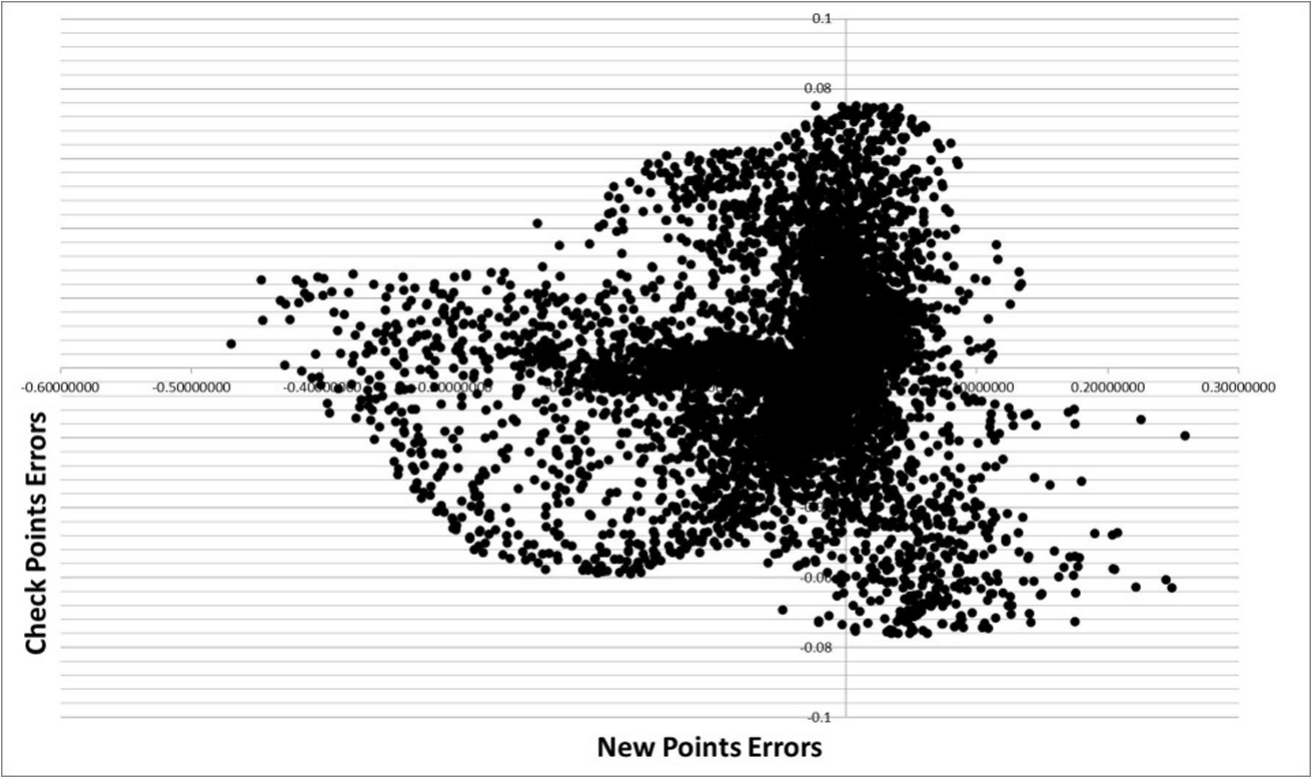
Comparisons between new points errors and check points errors.

Fig 9 shows the relationship between errors at new points and check points. This analysis indicates that there is no strong trend between these two quantities. Table 3 shows the Pearson product-moment correlation coefficients (PPCC) among the four parameters, which is formulated as

$$\rho_{X,Y}=\frac{cov\left(X,Y\right)}{\sigma_{X}\sigma_{Y}},$$ (18)

where *cov*(*X*, *Y*) is the covariance of *X* and *Y*, and *σ*_*X*_ and *σ*_*Y*_ are the standard deviation of *X* and *Y*, respectively. The correlation coefficient indicates a weak correlation between errors at new points and check points, in keeping with Fig 9.

**Table 3:** Pearson product-moment correlation coefficient among parameters.

	x	y	New Points Error	Check Points Error
**x**	1			
**y**	0	1		
**New Points Error**	0.00636	0.21588	1	
**Check Points Error**	-0.33681	0.32410	0.03341	1

### A real-world example

**Fig 10:**
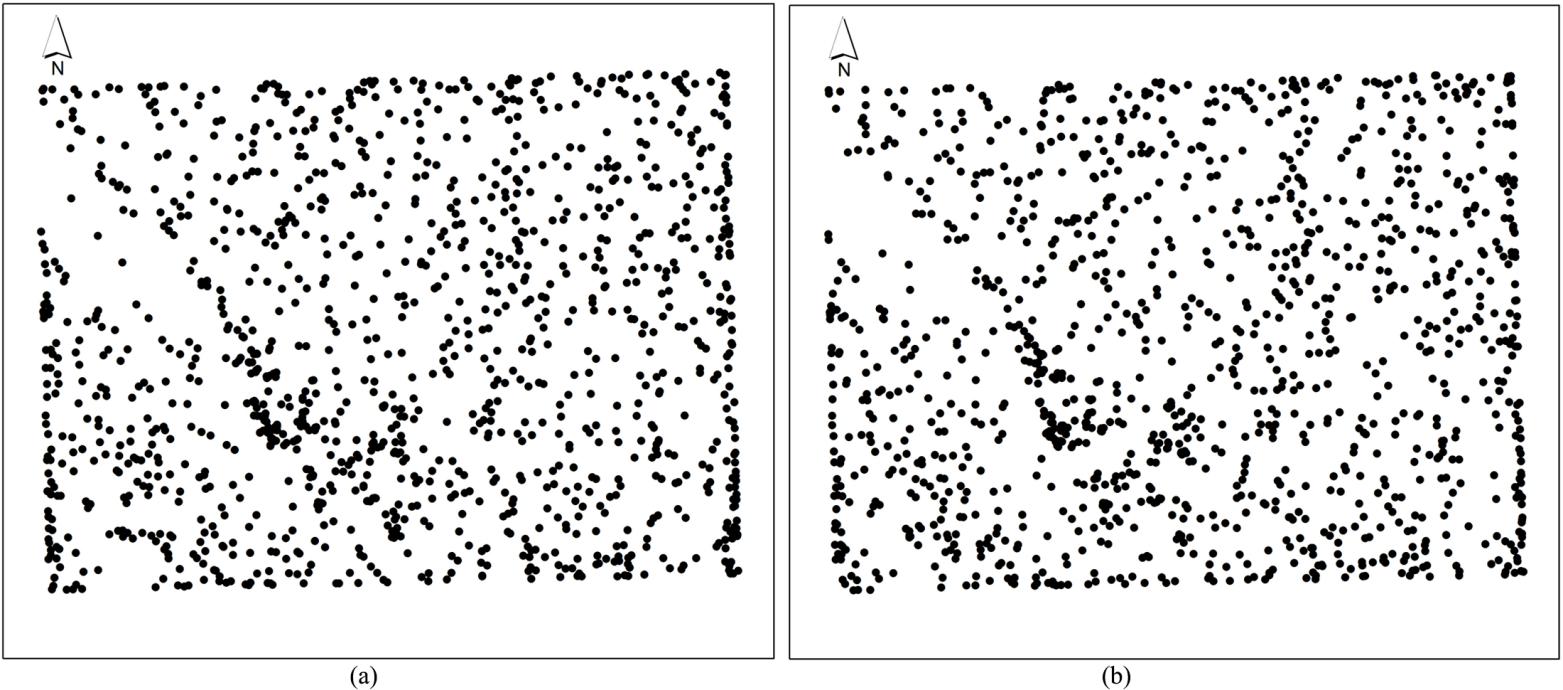
Location of elevation sampling points of Midu County. (a) Points for DEM construction, (b) Points for inspecting DEM accuracy.

**Fig 11:**
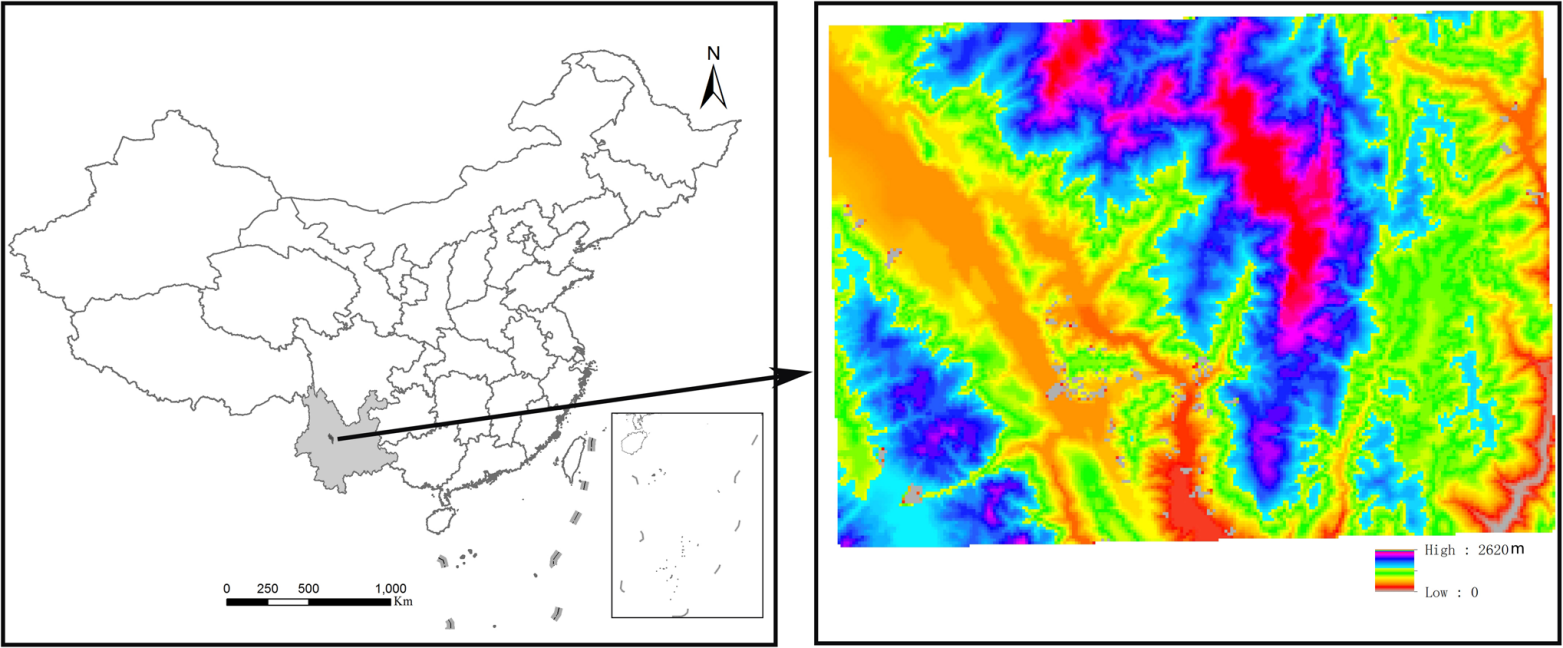
Location and topography of Midu County.

1209 elevation data points (Fig 10A) taken as original points and 6209 check points (Fig 10B) were randomly taken from a 1:50000 contour map to construct a DTM using QIN method, TIN method, SPLINE and KRIGING. The errors about these methods were comparatively analysed. The study area is located in Midu County, in the centre of Yunnan Province, China (Fig 11). The elevation ranges from 0 to 2620 meters and is suitable for a comparative study of DTM accuracy.

**Fig 12:**
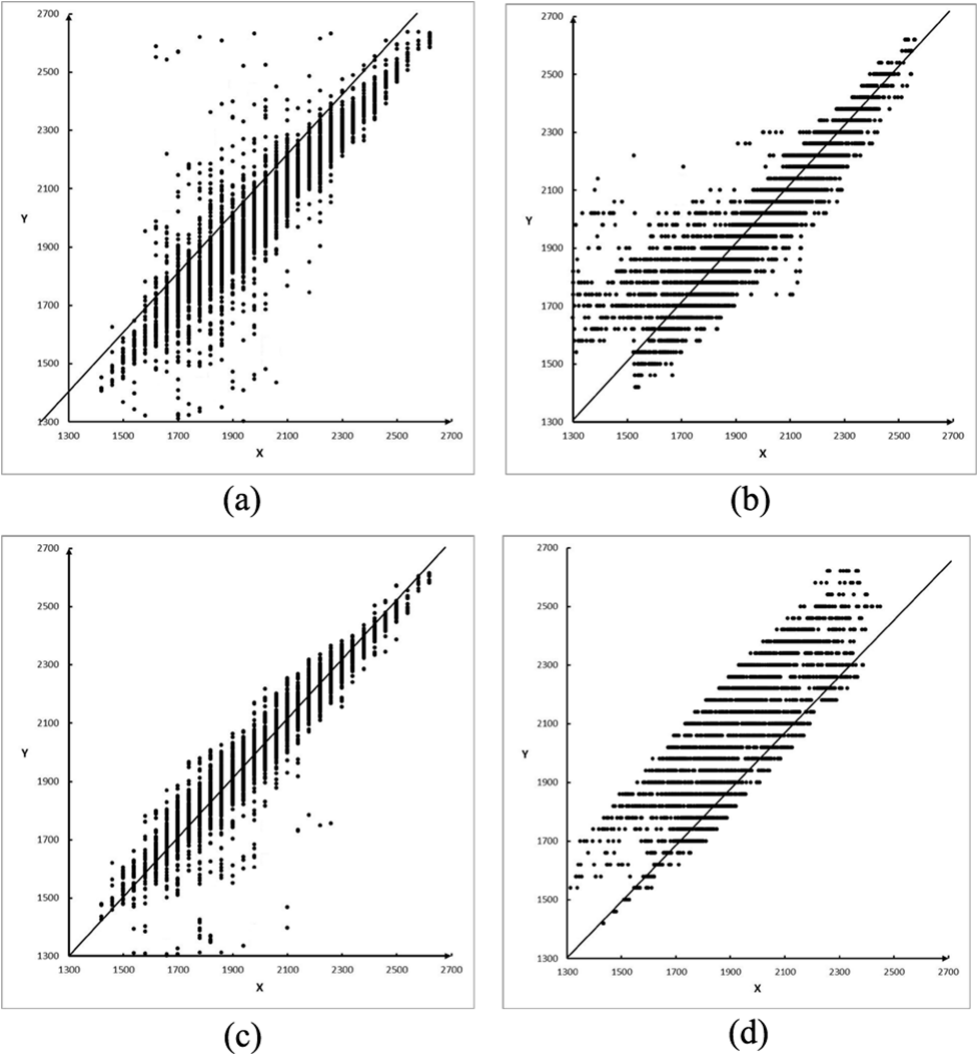
Comparisons between simulation results and observed data. (a) SPLINE, (b) KRIGING, (c) TIN and (d) QIN.

Fig 12 shows the relationship between the heights of the interpolated points and the corresponding observed heights of the points. This analysis indicates that the QIN method has the highest interpolation accuracy. The results from the SPLINE simulation appear more discrete and are lower than the line *y = x* overall. The KRIGING method has fewer points than SPLINE and tends to overestimate the elevations of lower check points. The TIN method has very few discrete simulation points and overestimates the elevations of lower check points even more than the KRIGING method does. The QIN method has the best result, but there are also some discrete simulation points.

## Discussion

The numerical test demonstrates that the accuracy of the QIN method is lower than that of the SPLINE method but higher than that of the KRIGING and TIN methods. In the real-world example, the accuracy of a QIN is the highest. The strategy of sampling discrete points, including original data points and check points, is the main reason why the QIN method does not achieve the highest accuracy in the numerical tests.

According to sampling theory, if there is a profile that can be expressed as a series of sine and cosine waves and is long enough to represent local terrain, a maximum frequency *F* can be resolved in the dataset. If the sampling interval is more than *1/(2F)* along the terrain profile, the DTM cannot be reconstructed completely. Such rigorous sampling rules were not used in either test conducted. However, under these sampling conditions, the QIN method is also a better choice for DTM construction.

Once the QIN model is built, the classical methods of digital terrain analysis can be applied based on this model, which has the same topology as a gridded DTM. However, the QIN model has huge computational and storage costs because it must generate a series of new data points and restore them for matrix structure construction. In the real-world test, there were 12,413 new data points generated, which was 10 times the number of original data points. To resolve this problem, two alternatives can be considered: 1) storing no new data points in data files, or 2) dividing the original data points into several clusters and constructing a QIN model based on each cluster, then integrating these QIN models. This is a direction of future research.
